# Editorial: Innovative strategies in overcoming glioblastoma: advancements in treatment and research

**DOI:** 10.3389/fonc.2026.1886865

**Published:** 2026-06-17

**Authors:** Raphael Bertani, Fabio Torregrossa, Felipe Salvagni

**Affiliations:** 1 Department of Neurosurgery, Hospital das Clínicas, University of São Paulo, São Paulo, SP, Brazil; 2 Neurosurgical Unit, Department of Biomedicine, Neurosciences and Advanced Diagnostics (BiND), University of Palermo, Palermo, Italy; 3 Department of Neurosurgery, Imigrantes Hospital e Maternidade de Brusque, Brusque, SC, Brazil

**Keywords:** biomarkers, blood brain barrier (BBB), computational oncology, drug delivery, glioblastoma, immunotherapy, liquid biopsy, tumor microenvironment

## Introduction

Glioblastoma (GBM) is still the most common and lethal primary malignant brain tumor in adults. Median overall survival hovers around 15 months, the same figure cited two decades ago when the Stupp protocol was published (1, 2). Tumor-treating fields remain the only addition to standard care that has shown a survival benefit in a phase III trial (3); every other major candidate has either failed or stalled in early-phase development. The reasons are well-rehearsed: genomic and epigenomic heterogeneity, an immunosuppressive microenvironment, the blood-brain barrier (BBB), and the diffuse, infiltrative biology of glioma cells. What is harder to admit is how often the field has interpreted preclinical signal as imminent clinical change, and how rarely that interpretation has held up.

This Research Topic was assembled with that history in mind. The Research Topic includes 17 articles spanning original research, systematic reviews, narrative reviews, a mini-review, and a bibliometric analysis, with contributing groups from four continents. We have organized the work along four axes: Molecular targets and the tumor microenvironment, Biomarkers and liquid biopsy, Novel therapeutic delivery and modalities, and Computational and genetic approaches. We aimed to read the contributions as parts of an argument rather than as simple summaries.

## Molecular targets and the tumor microenvironment

A claim that should by now be uncontroversial threads through the molecular contributions in this Research Topic: GBM resistance is not the product of a single driver but of a network of pathways operating within an actively immunosuppressive microenvironment. The therapeutic implication is that monotherapies aimed at single nodes will continue to disappoint, and that the more interesting question is which combinations of axes can be modulated together without unacceptable toxicity.

Three contributions illustrate insights regarding immune evasion. Ioannou et al. review the STING pathway as an immunotherapeutic target, covering cyclic dinucleotide agonism, repolarization of tumor-associated macrophages toward an M1 phenotype, and the potential to restore STING activity in epigenetically silenced tumors using demethylating agents such as Zebularine, and they are appropriately balanced about why none of this has yet translated, with systemic toxicity, rapid clearance, and poor BBB penetration keeping STING agonists almost entirely in preclinical territory. A possibly complementary mechanism is described by Gou et al., who show that CD58 drives PD-L1 upregulation, with downstream secretion of immunosuppressive chemokines (CCL5, CXCL9, CXCL10) and CD8+ T cell exhaustion; the mechanistic work is solid, though whether CD58 blockade can convert the cold immune phenotype of GBM into something more responsive will require more than the preclinical material currently available. Yin et al. take the question into the less-explored epigenetic territory and examine crotonylation, a histone post-translational modification in which a crotonyl group is added to lysine residues, much like acetylation, driving oncogenic transcriptions and promoting more aggressive tumor phenotypes. They present a crotonylation risk score that inversely correlates with glioma grade, with high-risk patients showing elevated TAM infiltration and resistance to checkpoint blockade, plausibly mediated by suppression of CXCL1 expression as crotonylation declines.

Three further contributions broaden the frame from immune evasion to the wider context of signaling and metabolism. Sagerer et al., in a mini-review of focal adhesion kinase signaling (FAK), make a point that generalizes well beyond the enzyme itself: the kinase behaves very differently in GBM, where pathway redundancy blunts the impact of monotherapy, than in NF2-mutant meningiomas, where loss of tumor suppressor moesin-ezrin-radixin-like protein (merlin) produces a synthetic lethal dependency. A target detected in a sequencing report is not the same as a functional dependency: a timely warning that trial designs ignoring this distinction may produce costly negative studies. The metabolic dimension is taken up by Zhang et al., whose integrated analysis of TCGA and CGGA transcriptomic data identifies fatty acid oxidation as an axis sustaining M2 macrophage polarization, with LGALS1, PLA2G5, and FABP5 emerging as markers of a high-risk immunosuppressive state across independent cohorts. Shen et al. add a tumor-suppressor counterweight, characterizing GADD45G through bulk and single-cell RNA sequencing as a brake on EMT-like invasion programs whose expression falls progressively in higher-grade tumors. None of these targets is, on present evidence, an obvious target for drugs, and the gap between a transcriptomic signature and a clinically deployable assay is no small hurdle. What these contributions do collectively, and usefully, is sharpen the map of where combination strategies might be designed from a biological viewpoint, not merely reframing what already exists.

## Biomarkers and liquid biopsy

Monitoring GBM biology without repeated surgery is a goal for all of us, but it has not yet been achieved. Three contributions in the Research Topic take complementary approaches to that problem, and read together they outline what a multi-layered diagnostic framework might eventually look like, while making clear that none of the components is yet ready for routine practice.

The most direct attempt at non-invasive detection comes from Wu et al., who show that chromatin accessibility patterns derived from plasma cfDNA can serve as an epigenetic classifier of glioma subtypes, using transcription factor-binding signatures such as MAZ, Myc-associated zinc finger protein, a transcription factor regulating proliferation and metabolism genes, whose chromatin binding footprint differs between low-grade and high-grade disease. The conceptual move from genotype to chromatin state is genuinely useful. The practical limitation, which the authors do not downplay, is that plasma cfDNA concentrations in patients with brain tumors are typically low, and the setting in which non-invasive detection would matter most (early or low-volume disease) is also the setting in which achieving sensitivity is most difficult. That ceiling is what motivates the systematic review and meta-analysis by Pérez-Alfayate et al., whose pooled estimates show CSF detection rates of approximately 82% versus 16% in plasma, with molecular concordance with tissue of nearly 90%, particularly for IDH1 mutations. The CSF advantage is not a new claim, but the quantitative confirmation is important. What is often glossed over, and worth keeping in view, is that lumbar puncture is not interchangeable with venipuncture, and ambitions of frequent serial monitoring should be calibrated to that clinical reality.

A different layer of the same problem is addressed by Xu et al., who use nontargeted tissue metabolomics to derive an 11-metabolite panel that distinguishes IDH-mutant GBM, IDH-wildtype GBM, and non-tumor brain tissue with reasonable accuracy in a Random Forest model, alongside a separate prognostic model based on cholic acid and citrulline that reaches an AUC of 0.942 at 84 days post-resection. The numbers are encouraging, and the underlying biology, that distinct metabolic fingerprints accompany distinct molecular subtypes, seems plausible. The cohort is small (which may invite overfitting of AUC), and the model has not been externally validated, which is the usual ceiling for tissue metabolomics studies of this type, and the contribution is best read as proof of concept rather than as a deployable assay. Taken together, these three papers describe a diagnostic pipeline that does not yet exist as a single clinical tool: a chromatin-aware plasma layer for screening, a CSF layer for molecular analysis when neurologically feasible, and a metabolic layer for tissue-level subtype refinement. The path from this conceptual framework to anything resembling routine practice is yet to be paved.

## Novel therapeutic delivery and modalities

The BBB remains the central pharmacokinetic problem in GBM, and a recurring theme across the delivery contributions is that a drug that cannot reach its target cannot be evaluated for efficacy; delivery is not a downstream concern but a primary one. The strategies represented in this Research Topic range from minimally invasive to frankly device-driven, and they share an honest acknowledgment that physics- and engineering-based circumventions of the BBB are now serious candidates rather than fringe alternatives.

Among the energy-based modalities, Medeiros Netto et al. review minimally invasive laser and light-based therapies, including laser interstitial thermal therapy (LITT), photodynamic therapy (PDT) with photosensitizers such as 5-ALA, and related photothermal techniques, across 23 studies, finding suggestive signals of survival benefit and tumor mass reduction at acceptable safety, with a separate observation that LITT may enhance tumor immunogenicity in ways that could synergize with checkpoint inhibition. However, the dominant message of the review is methodological: heterogeneity in study design, light source, photosensitizer, and outcome definition is sufficient to preclude pooling of efficacy estimates, and standardization is a precondition for any large trial that aims to be interpretable. The risk of distant dissemination associated with PDT, separately, deserves explicit mention in patient counseling rather than a footnote. Sun et al. advance a complementary non-invasive approach, reviewing static magnetic field therapy (SMF) as a potential adjunct in glioma, with proposed mechanisms including interference with EGFR signaling, mitotic spindle disruption, anti-angiogenic effects on the TME, and modulation of magnetic nanoparticle distribution to improve targeted drug delivery across the BBB. As an idea, SMF is appealing because of its non-invasive profile; as a clinical proposition, the evidence remains overwhelmingly preclinical, and the leap to a human trial design that yields interpretable endpoints has not yet been convincingly made.

Direct circumvention is addressed by Berlet et al., who describe an implantable, 3D-printed intracerebral osmotic pump for convection-enhanced delivery, with reported parenchymal distribution extending up to 18 mm beyond the resection cavity. CED bypasses the BBB by definition, and the proof-of-concept data are encouraging; the clinical concerns are familiar to other implanted CNS devices, including infection, mechanical failure over time, and the difficulty of achieving uniform drug distribution in a geometrically irregular surgical bed, and none is disqualifying. Each will need prospective data before this platform can be thoroughly assessed. Biological delivery is represented by Yu et al., who examine oncolytic virotherapy with Newcastle Disease Virus, identifying the antiviral gene OASL as a host factor that aggravates rather than restrains NDV-induced necroptosis; knockdown of OASL attenuated this process in their experimental system, raising the mechanistically coherent possibility that OASL modulation could maximize oncolytic efficacy. Oncolytic virotherapy in GBM has a long history of preclinical promise and clinically unmet expectations, with this contribution sitting firmly on the preclinical end of that arc.

One contribution that lies at the interface between delivery and the neurological cost of treatment and, in our view, is among the more clinically actionable papers in the Research Topic. Costa Santos et al. characterize Wallerian degeneration of the corticospinal tract following multimodal high-grade glioma treatment, with an incidence of up to 6.8% in their cohort and the capacity to produce severe hemiparesis. Successful oncological treatment will not be a satisfactory endpoint if the patient is left with a major motor deficit, and radiotherapy planning should take this into consideration. Diffuse tensor imaging (DTI)-based tractography is technically available at most academic centers, and although larger-cohort validation is appropriate, the operational message is clear.

## Computational and genetic approaches

Where the molecular and delivery contributions in this Research Topic focus on what to do, the computational and genetic contributions address how decisions are made, at the bedside, in counseling, and in the design of trials. The connecting theme is that the standard of care still relies on population-level estimates and one-size-fits-all assumptions that do not match the heterogeneity documented by molecular work.

The bedside problem is taken up most directly by Kostelich et al., who apply a reaction-diffusion mathematical framework, the ASU-Barrow model, to standard surveillance MRI data, generating individualized tumor growth and treatment-response scenarios at 2- to 3-month intervals. The intent is bedside, not academic: to give patients and families a data-driven range of plausible trajectories rather than a single guess, in a clinical situation where guesses are often what is offered. The framing as ensemble-based forecasting is reasonable, and the authors are open about the limitations of any model that assumes a biological homogeneity it cannot verify; external validation in prospective cohorts is the necessary next step. Recurrent GBM counseling, as currently practiced, often consists of population-level survival ranges that are accurate but unhelpful at the individual level, and the clinical need this work addresses is real.

Another blind spot in the current literature is addressed by Phabphal et al., who use whole-exome sequencing to identify candidate gene mutations associated with seizure susceptibility in patients with astrocytoma, with IDH1 and NMDA receptor-related genes emerging as drivers. Tumor-related epilepsy is a chronically underinvestigated topic in neuro-oncology relative to its actual impact on patients, who often rate seizure control above other endpoints in their day-to-day quality of life. By describing genetic determinants of epileptogenicity that operate independently of size and location of tumors, the groundwork for precision-guided seizure prophylaxis can be laid down.

The Research Topic closes with a bibliometric contribution from Hong et al., who analyze more than 9, 700 publications from 2020 to 2024 and document the field’s pivot toward targeted drug delivery and immunotherapy, with “immune infiltration” showing the strongest recent citation burst and China and the United States leading in publication volume. Whether that volume corresponds to clinical progress is a separate question, and bibliometric trajectories should be read as a description of attention rather than impact.

## Conclusion and future directions

The contributions in this Research Topic show that efforts against glioblastoma are underway across multiple fronts: molecular target identification, immune evasion mechanisms, delivery platforms, liquid biopsy assays, and computational decision support. The breadth is real. So are the gaps.

Liquid biopsy panels, whether based on CSF ctDNA, cfDNA chromatin accessibility, or tissue metabolomics, need prospective validation in trials that connect them to actual treatment decisions. Delivery platforms, from CED osmotic pumps to PDT protocols and SMF-modulated nanoparticle distribution, require standardization and phase I/II safety data in cohorts sufficiently homogeneous to enable interpretation of efficacy signals. The molecular targets emerging from bioinformatics and single-cell work, including GADD45G, the CD58/PD-L1 axis, crotonylation modulation, context-specific FAK inhibition, and lipid metabolism signatures, require functional validation in models that more closely resemble the clinical disease. And the neurological costs of treatment, of which Wallerian degeneration is only one example, need to be incorporated into the standard of care rather than absorbed as background damage.

The diversity of approaches in this Research Topic argues against siloed work and in favor of integrative programs that bring molecular biology, engineering, computation, and clinical practice into the same room. The path from bench to bedside in GBM has been longer than expected. The work captured here suggests a field that is not only honest about that distance but also more willing to subject its own claims to critical scrutiny with a resolve that these efforts translate into outcomes that matter for patients in the near future.
